# Frontal Impact Energy Absorbers for Passenger Cars

**DOI:** 10.3390/s24206563

**Published:** 2024-10-11

**Authors:** Filip Dąbrowski, Zuzanna Grzejszczyk, Cezary Rzymkowski, Piotr Wiśniewski

**Affiliations:** 1 Łukasiewicz Research Network—Automotive Industry Institute (Łukasiewicz–PIMOT), Jagiellonska 55 Street, 03-301 Warsaw, Poland; filip.dabrowski@pimot.lukasiewicz.gov.pl (F.D.); piotr.wisniewski@pimot.lukasiewicz.gov.pl (P.W.); 2 Institute of Aeronautics and Applied Mechanics, Faculty of Power and Aeronautical Engineering, Warsaw University of Technology, 00-665 Warsaw, Poland; cezary.rzymkowski@pw.edu.pl

**Keywords:** crash box, energy absorber, passive safety, accident prevention, crashworthiness

## Abstract

Road accidents cause considerable losses to road users and to society. The steady increase in the number of vehicles leads to increased traffic volume. Therefore, there is a real need to improve passenger safety by developing passive safety systems. This article presents the results of experimental tests of structures absorbing kinetic energy, which could be used in the front section of a vehicle in order to reduce the consequences of passenger car head-on collisions. A number of crash tests of selected structures were conducted under various load conditions. An analysis was carried out of parameters enabling the authors to assess the level of energy absorption by the absorbers made, and compare these to absorbers available on the market. The tests carried out made it possible to determine energy absorption capability of the crash boxes prepared and to identify a structure exhibiting the most advantageous properties from the point of view of its prospective use. Of all of the absorbers analysed, in the context of energy absorption, it was the absorber made of glass-fibre-reinforced polyphenylene sulphide that produced the most advantageous results. Nonetheless, favourable results were obtained for all of the structures tested.

## 1. Introduction

Road transport is one of the key sectors driving the growth of the national economy. As much as 8% of GDP and 13.5% of annual transport is based on the automotive industry. It must be noted, however, that despite the beneficial impact of the automotive sector on the country’s economy, the development of this sector brings with it a tremendous threat to health and life arising from road accidents, the effects of which are felt not only in people’s individual lives, but also in the social sphere. The high costs of the consequences of traffic collisions and road accidents have a negative impact on the economic interests of the state and of its citizens [1,2]. According to data published by the WHO, in 2021 alone, there were ca. 1.19 million road traffic deaths [3]. Road accidents cause considerable economic losses to the victims and their families as well as entire nations. The afore-mentioned losses arise from the cost of treatment, lost productivity in respect of those killed or disabled by their injuries, and in respect of family members who often need to take time off work or school to care for injured relatives. Road accidents cost most countries ca. 3% of their gross domestic product. In Poland, in 2021, the unit cost of a fatality was PLN 2.6 million, and that of a seriously injured victim was PLN 3.5 million [2].

**Figure 1:**
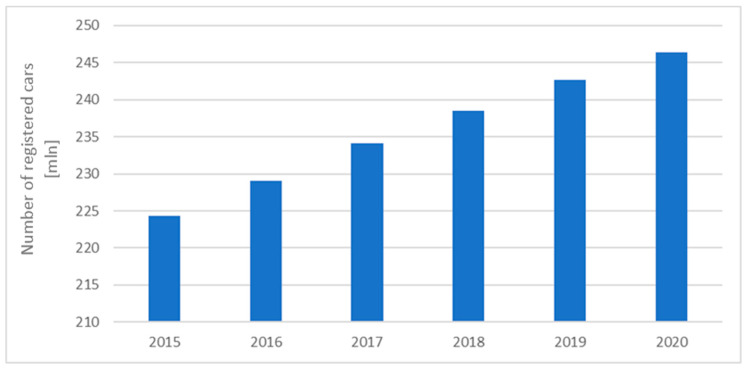
Number of cars registered in Europe between 2015 and 2020 [ACEA Report].

**Figure 2:**
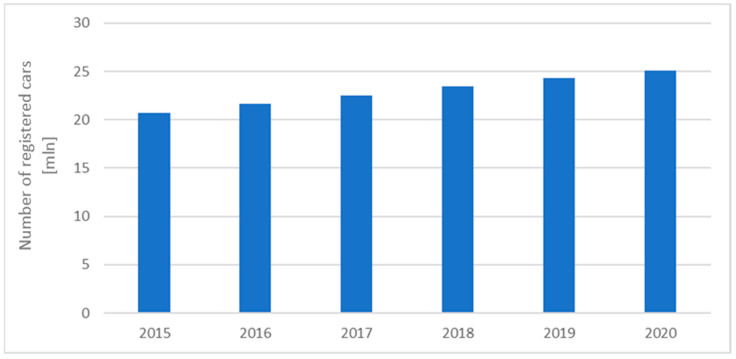
Number of cars registered in Poland between 2015 and 2020 [ACEA Report].

Over the past few years (2015–2020), the number of passenger cars in Europe has increased by more than 9.8% (see Figure 1); a similar trend is present in the Polish car market, where the number of registered vehicles has been rising steadily (see Figure 2).

As road safety requirements are increasingly stringent, car manufacturers are having to resort to ever more sophisticated technologies relating to vehicle strength, durability, and safety. However, road accidents continue to kill and injure numerous road users—in the year 2021, the highest fatality rate per 100 accidents in Europe was recorded for Poland (9.8) and Bulgaria (9.2). As for the injured persons rate, Luxembourg (135.7) and Italy (134.8) were at the top of the list. According to the data provided by Poland’s National Police Headquarters, for years now, a consistent trend has existed whereby most people die as a result of head-on collisions relative to other types of vehicle crashes (Table 1) [4].

Given the still high number of fatalities in the past few years (ca. 2000–3000 fatalities annually) and a slightly decreasing number of injured persons (24,743 persons in 2022) [4], it is important that new measures should be urgently implemented to improve road safety. Such measures should focus on road user education, improvements in road conditions, and improving vehicles.

The automotive safety discipline is concerned with such aspects as improvements in vehicle design and vehicle features that can help minimize negative impacts of road traffic. Active vehicle safety is becoming increasingly important in preventing accidents, while passive safety contributes to reducing fatalities and serious injuries that can lead to disability [5,6]. Improvements to individual components used to enhance crashworthiness can be made both by modifying or developing a new vehicle structure, and by using, in vehicle design, innovative materials and structures that absorb kinetic impact energy to a larger extent than the ones now utilized [7].

**Figure 3:**
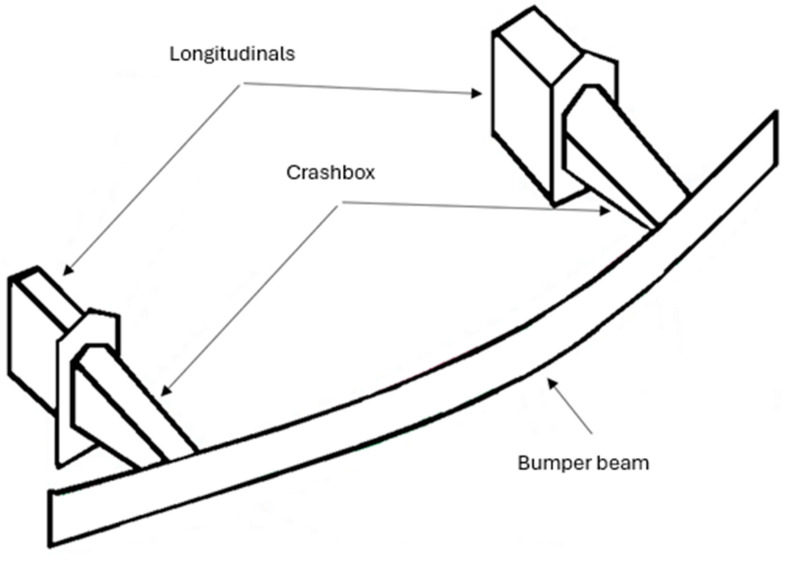
General schematic of front section components of a car body.

One of the components commonly used in cars is energy absorbers or crash boxes, installed between bumper beams and longitudinals. Figure 3 presents a general schematic of the placement of the absorbers in the vehicle. They are primarily designed to absorb impact energy up to speeds of ca. 20–25 km/h [8]. The authors carried out a number of strength tests on crash boxes that were developed as part of project No. 5/Ł-PIMOT/CŁ/2021, referred to as a design of frontal impact energy absorbers for passenger cars. The tests were intended to experimentally verify the energy-absorbing properties of crash boxes.

In the project, the focus was primarily on the passenger safety aspects of using energy absorbers in vehicles, considering them as a crucial element of protection in the event of a collision or accident. The priority was to understand and optimize the properties of these components in terms of their ability to absorb collision energy, thereby minimizing the risk of injury to passengers.

Economic aspects, such as production and operational costs, were deemed secondary in this case. The aim was to identify the most efficient structures in terms of their energy-absorbing capabilities, even if this would lead to higher production costs. This research strategy is justified in situations where safety is a key requirement.

## 2. Description of Energy-Absorbing Structures

The function of energy-absorbing materials used in vehicles is to dissipate kinetic impact energy primarily through friction and plastic deformation. At the same time, the rate at which deformations occur should keep the force impulse below the critical level, i.e., a level that can generate significant load to which the vehicle passengers are subjected [9,10]. In the automotive industry, profiles constituting multi-cell structures of various shapes are used for this purpose. Materials most often used in energy-absorbing components include low-carbon steel, aluminium alloys, and metallic and non-metallic foams [8,11,12,13,14,15,16]. Generally, they are marked by good plastic properties [11]. The research results described in the publications provide general knowledge of the properties of the structures and materials used, but due to the different research methodologies used, the authors felt it necessary to carry out their own research based on their own methodology.

Energy-absorbing components should operate in such a way as to ensure irreversible energy conversion, so the kinetic energy of a moving object should be converted into work through plastic deformation in order to avoid accumulating potential strain energy [17,18,19,20,21,22,23].

Deformation work is equal to force multiplied by deformation distance. That is why, in order for a component to absorb a sufficient amount of energy and for a specific load not to be exceeded, an adequate, i.e., maximally long deformation distance should be ensured. This will allow the force value to be kept at a constant level, below a specific limit (e.g., determined in relation to the resistance of the human body) [17,18,19,20,21,22,23].

It should be noted that external dynamic loads may vary in terms of such characteristics as value, direction, or place of application. Energy-absorbing components should ensure a stable and repeatable deformation and absorb the required amount of energy irrespective of the above factors. Furthermore, attempts should be made to keep the mass of such a component to a minimum and, at the same time, to ensure maximum effectiveness of energy absorption [17,18,19,20,21,22,23].

Equally important are economic factors such as low cost and easy installation associated with such cost. These economic factors can be crucial from a business point of view and can have a significant effect on the dissemination of effective energy-absorbing solutions [10,17,18,20,21,22,23].

## 3. Research Methods

A number of crash tests were conducted to verify the energy-absorbing properties of selected structures/materials. The tests were conducted according to a specially developed procedure allowing the authors to obtain the data/information necessary for determining the strength characteristics and indicators needed to compare the test results obtained and assess the specimens tested.

### 3.1. Test Rig

**Figure 4:**
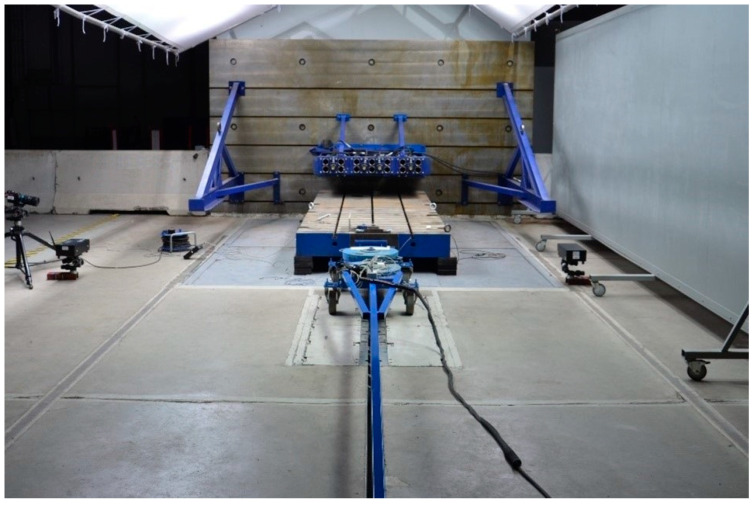
Test rig and the test sled.

The tests were conducted at a crash test rig in the Vehicle Safety Laboratory of the Łukasiewicz Research Network—Automotive Industry Institute. A test sled with a mass of 350 kg was used in the tests (Figure 4). In the front section of the sled, a flat plate was installed, perpendicular to the floor, which made it possible to mount the specimen to be tested. The body of the sled had installed on it a tri-axial acceleration sensor kit which, complete with a data acquisition system, recorded test sled acceleration during the tests.

The tests were filmed using a high-speed camera operating at 2500 frames per second, installed perpendicularly to the direction of sled impact.

### 3.2. Description of the Structures Tested

**Figure 5:**
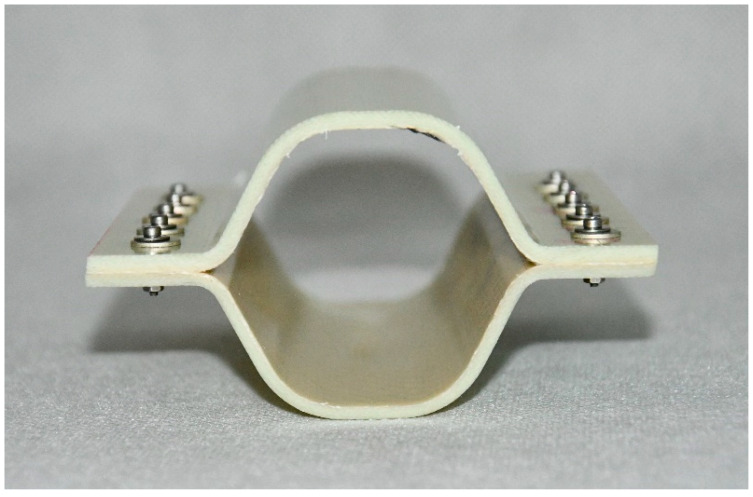
Cross-sectional view of the working element of the structures tested.

Based on an analysis of the current state of the art and solutions currently used in the market, desired properties were identified for crash boxes installed in the front sections of passenger cars. The main desired properties include the following:

- The length of the working part should be 130–140 mm;
- The cross-section of the working element should preferably be rectangular and have dimensions of ca. 120 × 60 mm (it is determined by the shape and size of longitudinals in modern vehicles) (Figure 5);
- Cuboid form.

Based on extensively described energy-absorbing structures in the literature, a shape similar to a honeycomb cell was selected, which exhibits unique mechanical properties—high strength relative to its mass. In a honeycomb-type structure, each cell acts as a sort of pillar, evenly distributing loads. The rounding of the corners of the structure further contributes to reducing stress concentration [14,22].

Another desired property was a maximally linear waveform of energy as a function of strain and maximum deceleration of the test sled achieved during the test—35 g–40 g, it being understood that this requirement is to be met in respect of crumpling for up to 80% of the length of the specimen. The rest of the absorber’s length (20%) was intentionally left uncompressed to provide sufficient space for the material during crushing. That allows for the controlled deformation of the remaining 80% of the absorber’s length, ensuring that the energy absorption capacity remains effective. An increase in deceleration values in the final deformation phase is acceptable.

**Figure 6:**
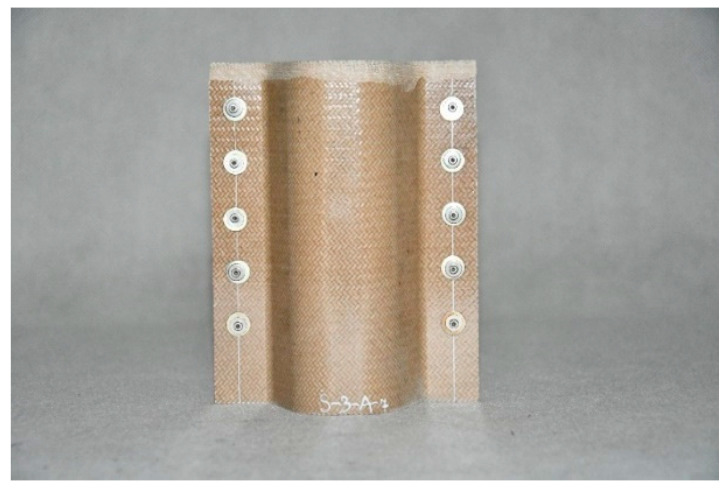
Glass-fibre-reinforced polyphenylene sulphide (PPS) absorber—WS-PPS.

**Figure 7:**
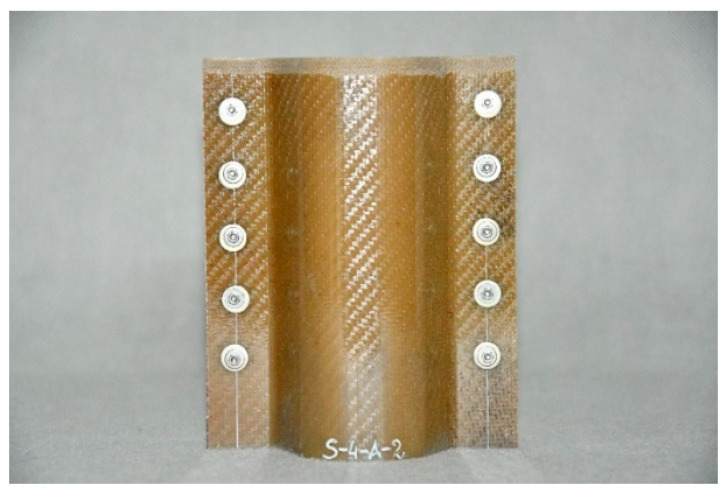
Glass-fibre-reinforced polyetherimide (PEI) absorber—WS-PEI.

**Figure 8:**
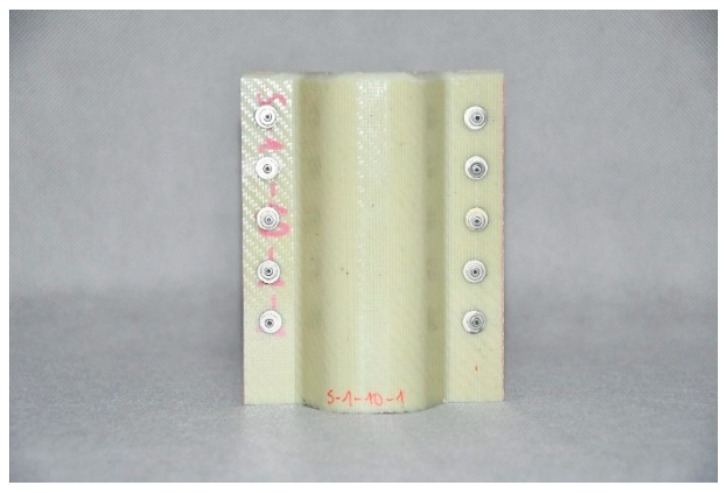
Glass fibre polyamide 6 (PA 6) absorber—WS-PA6.

**Figure 9:**
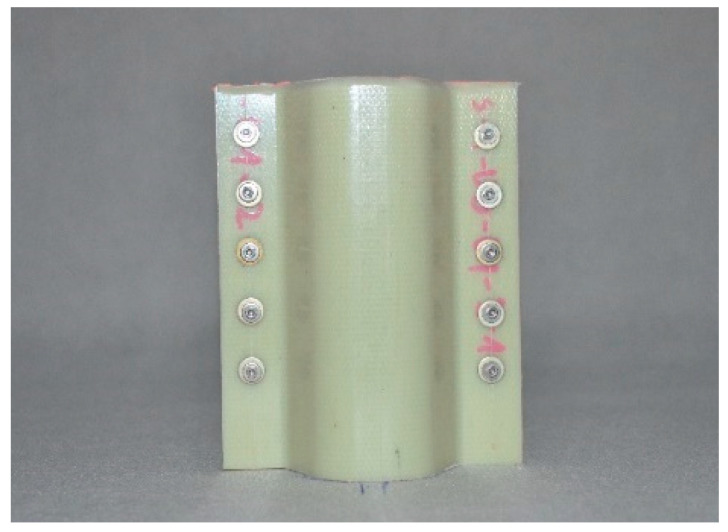
Glass fibre polyamide 6 (PA 6) absorber, with 85% of fibres running in one direction—WS-PA6-85.

Composite structures of identical shape were selected for testing; such a shape makes it possible to fit the structures to the longitudinals in typical passenger cars. The structures were made of various plastics, but contained the same reinforcing material, i.e., glass fibre. The specifications of the crash boxes selected for testing are set forth in Table 2, and the crash boxes are shown in Figure 6, Figure 7, Figure 8 and Figure 9. Since all of the connecting elements were identical, their impact on the research results was considered negligible, which allowed for the simplification of the comparative analysis and focus on other significant aspects of the study.

### 3.3. Methodology

The aforementioned crash boxes were tested in accordance with the procedure described in Section 3.1. The following test speeds were selected: 10, 16, 20, and 24 km/h. Before each test, the face of the front section of the sled had a test item mounted on it, i.e., a crash box. The sled was accelerated to a pre-defined speed. The speeding sled, with the crash box installed on it, struck a rigid barrier. Upon the impact of the crash box under test against the barrier, the data recording system was activated (capturing accelerations measured with a three-axial sensor system and high-speed cameras).

The acceleration components recorded were filtered using a CFC60 filter. Based on the filtered acceleration waveforms, a resultant deceleration waveform was plotted. As a result, the authors managed to plot a waveform of the inertia force of the sled, thus determining the amount of the energy dissipated by the crash box. Due to the cumulative error involved in using double numerical integration, it was decided not to determine the deformation of the absorber with this method. Absorber deformation was determined each time with the cinematographic method, using high-speed camera footage and dedicated specialist software. The software algorithm used a mechanism for tracking point tags on recorded video footage and, taking into account appropriate scaling data, the distance was determined between a tag on the moving sled and a stationary tag at the test rig for each frame of the recorded video. For the above reasons, the separation speed of the test sled was also determined with the cinematographic method.

**Figure 10:**
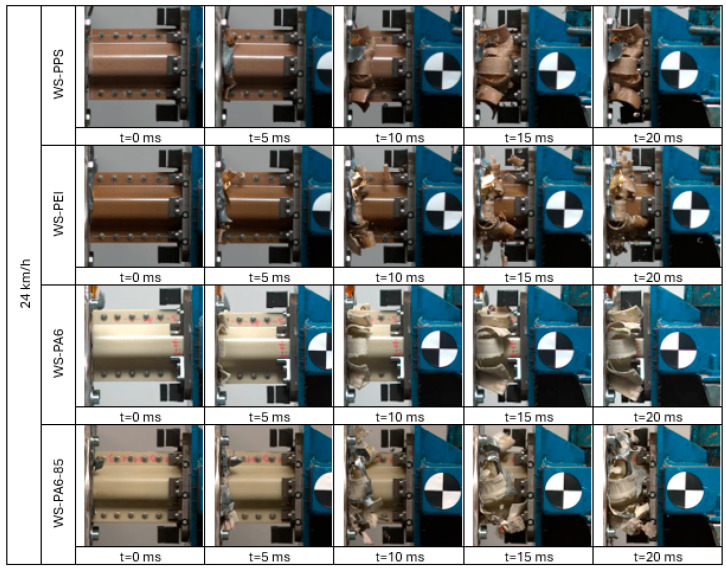
Crash boxes during tests at speed of 24 km/h.

Images obtained with the high-speed camera showing the objects at defined moments of the test are shown in Figure 10. 

## 4. Test Results

**Figure 11:**
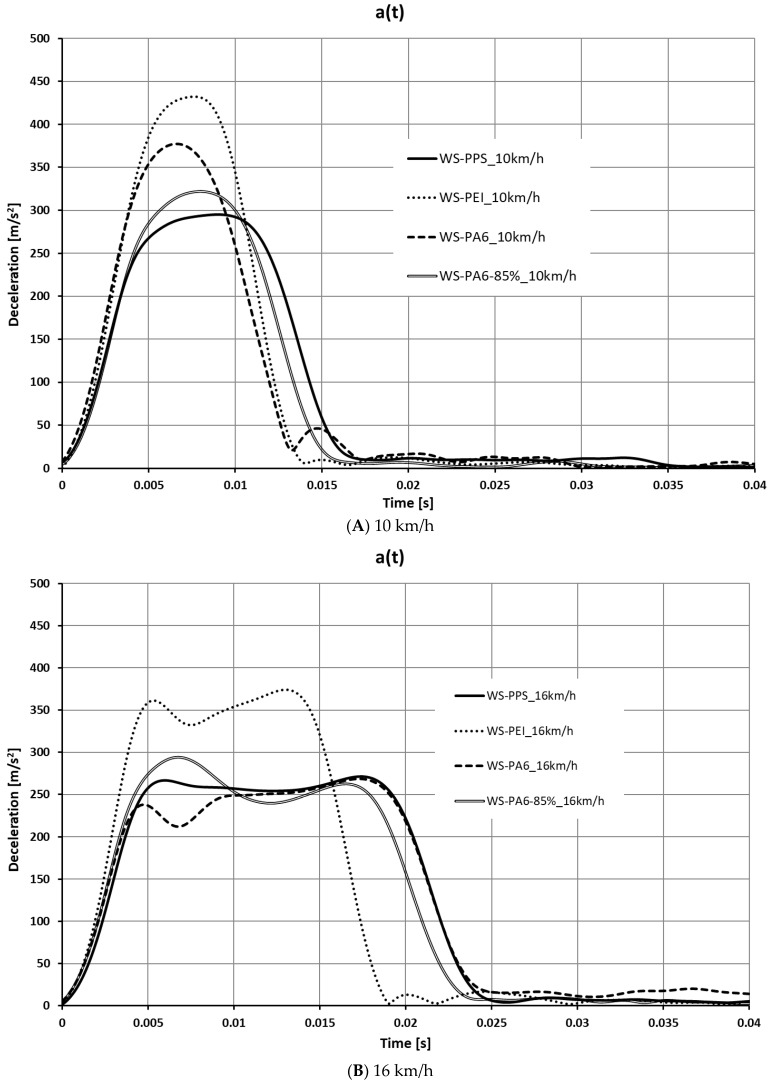

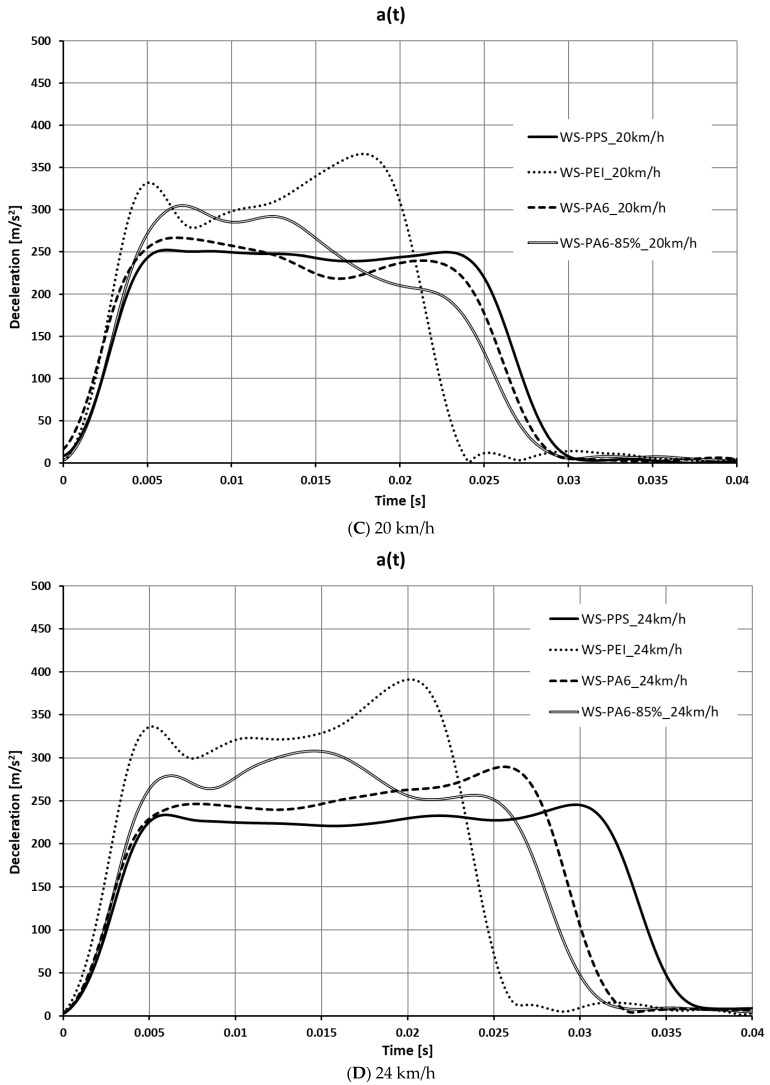
Deceleration as a function of time a(t).

**Figure 12:**
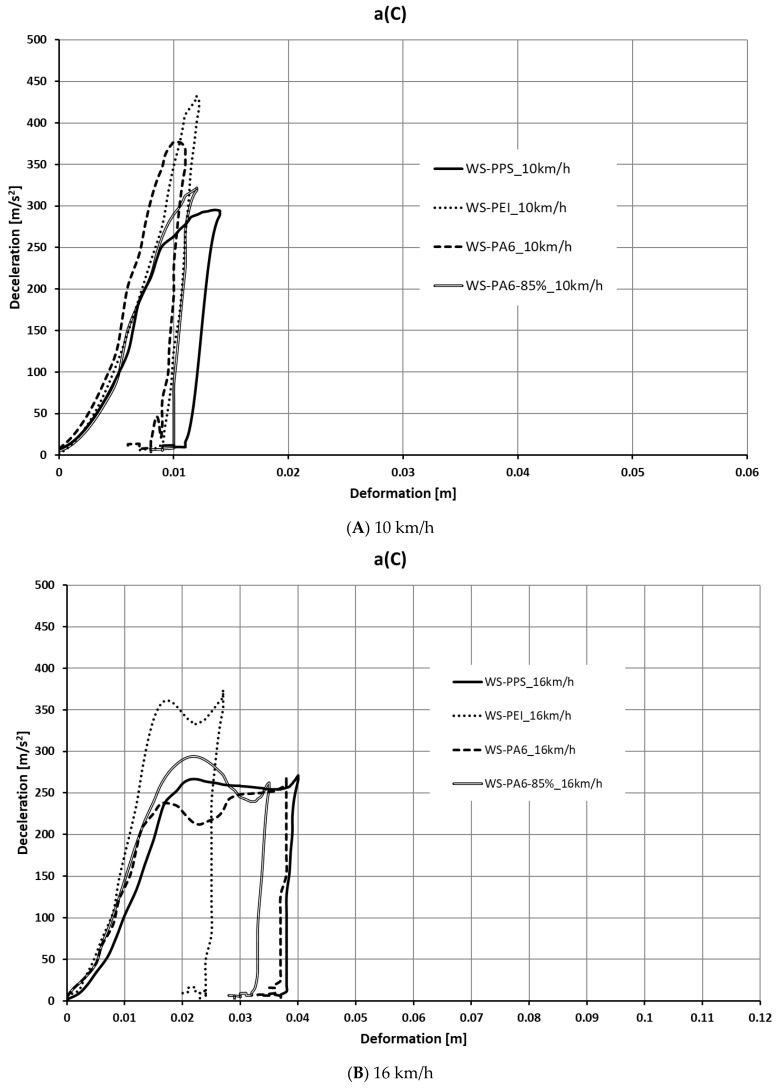

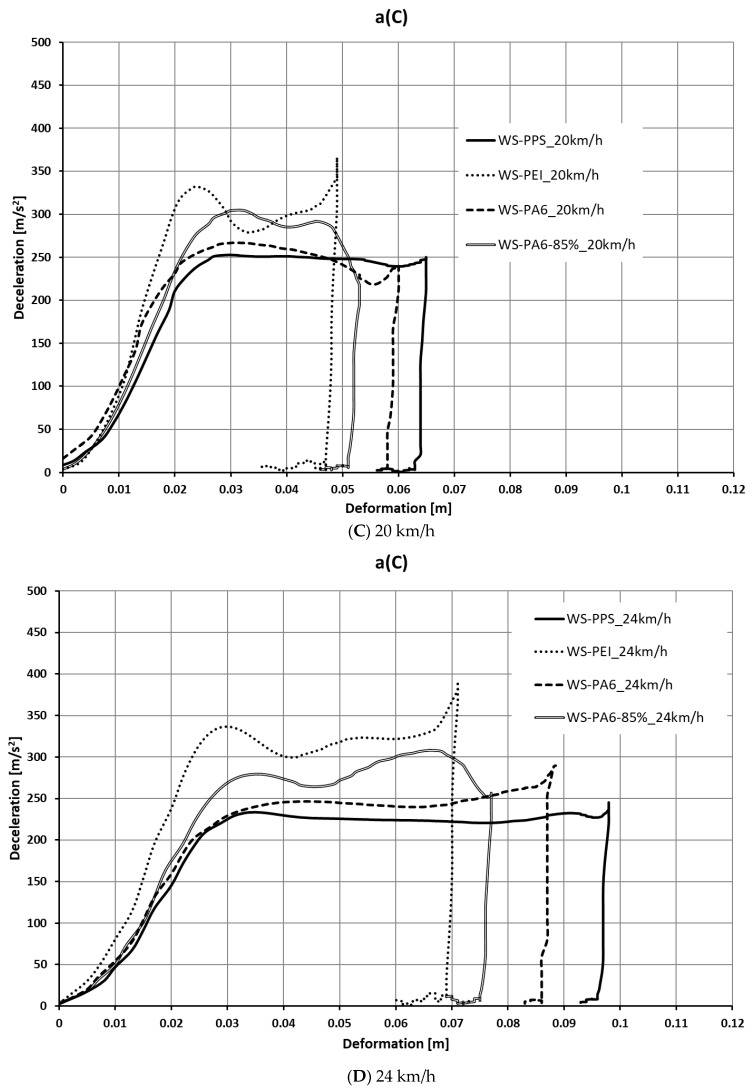
Deceleration as a function of deformation a(C).

**Figure 13:**
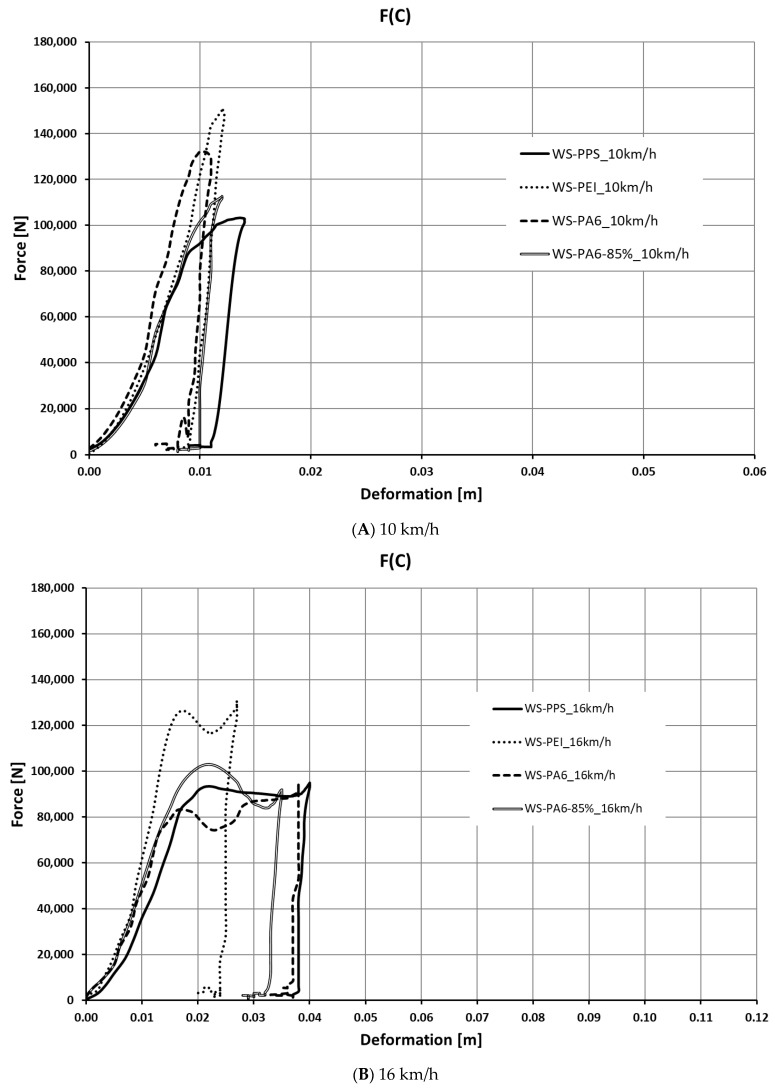

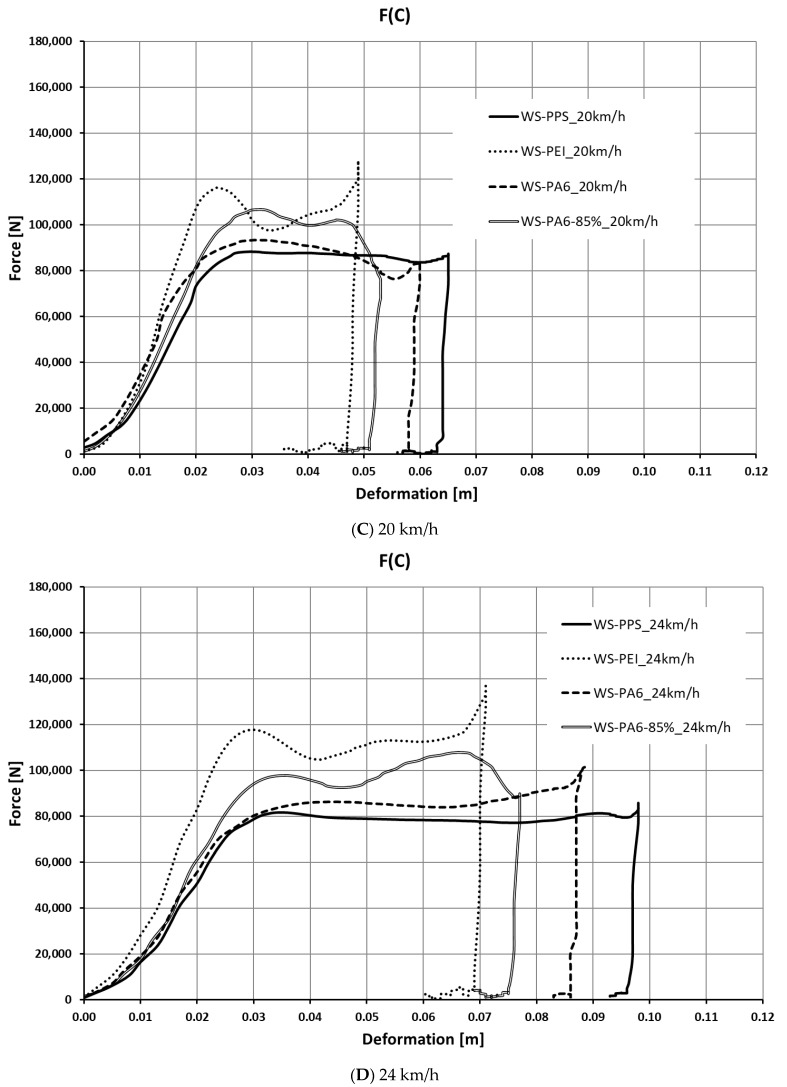
Waveform of force as a function of deformation F(C).

**Figure 14:**
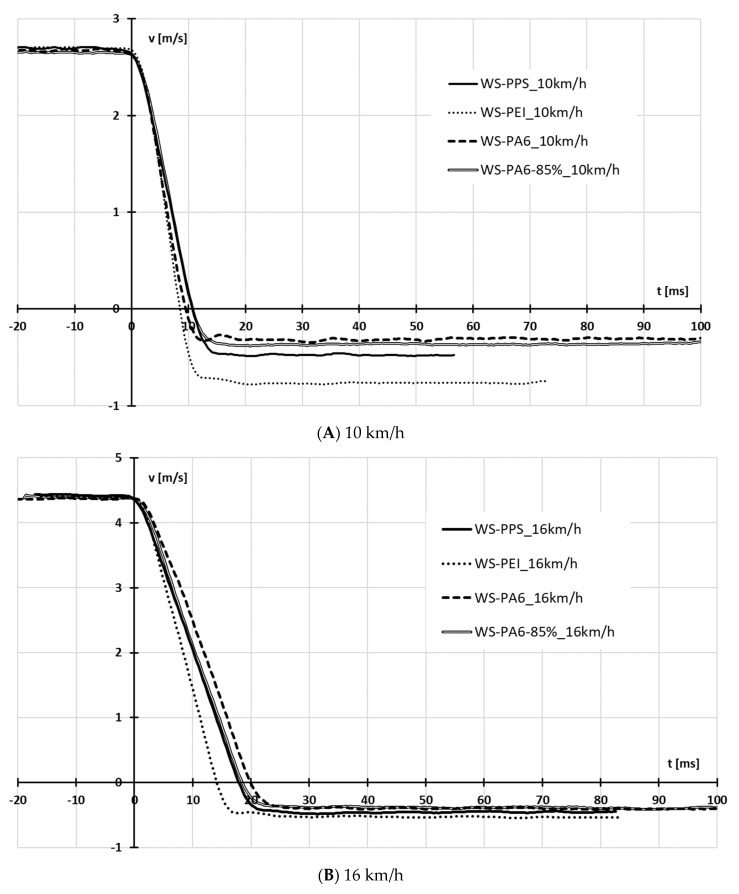

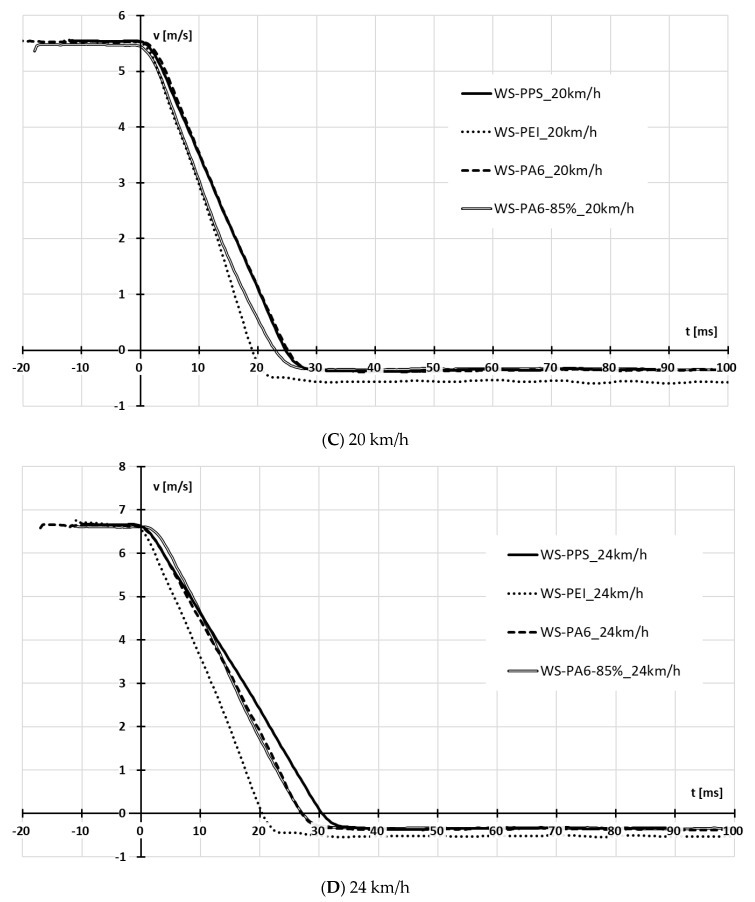
Waveform of speed during impact.

Based on the calculations performed, a number of characteristics were determined for the crash boxes discussed. The characteristics are shown in Figure 11, Figure 12, Figure 13 and Figure 14.

Based on an analysis of the deceleration waveforms as a function of time and deformation (Figure 12) it was found that the greatest deceleration was recorded for the structures labelled WS-PEI, and varied in the range of 37–43 g. At both 10 km/h and 24 km/h, the lowest deceleration was recorded for the WS-PPS structures. At speeds of 16 km/h and 20 km/h, the WS-PPS and WS-PA6 crash box generated similar, almost flat deceleration waveforms and values, slightly lower than the other crash boxes.

What is special with regard to the results presented is that the greatest deceleration values were obtained for the speed of 10 km/h. This was due to the fact that at low-impact speeds, for the most part, the absorbers were deformed elastically, and the kinetic energy of the sled during the first deformation phase, to a large extent, was used up to exceed the yield point.

Based on an analysis of the waveforms of force as a function of deformation, it was found that the highest values of force were obtained for the WS-PEI structure, with one of the smallest deformations occurring. The highest force values fell within the range of 120–150 kN, with the highest value recorded at a speed of 10 km/h. The lowest value of force, accompanied by the largest deformation, was recorded for WS-PPS.

As the analysis shows, in respect of higher speeds (20 km/h and 24 km/h), the structures made of WS-PPS and WS-PA6 are marked by a stable deformation process. The value of force and deceleration remains constant or changes to a slight extent, and, except for the last deformation phase, no abrupt changes of force or deceleration values occur. In the case of the WS-PEI structure, the deformation process is clearly less stable (at the beginning and the end of the deformation process, abrupt peaks occur).

Each of the tests was an elastoplastic impact; i.e., in addition to permanent deformations, there were also elastic deformations of the structures under test, which reversed after the force ceased to act upon the item tested. The coefficient of restitution *k* indicates a degree of elasticity of a collision and is defined as the ratio of the velocity of separation to initial velocity (*k* = 1—a perfectly elastic collision, *k* = 0—a perfectly plastic collision, 1 > *k* > 0—an elastoplastic collision). Table 3 sets forth the coefficients of restitution for each of the tests and data on the percentage share of separation energy in initial energy.

The highest elastic modulus values were recorded for collisions at 10 km/h. As the collision velocity increased, the values of restitution coefficient were observed to decrease. At 24 km/h, the structures exhibited almost identical elasticity—a slightly higher restitution coefficient was determined for WS-PEI.

In order to ensure more precise specimen parametrization, EA, SEA, MCF, PCF, and CLE indexes were determined, which enabled the authors to conduct an additional comparative analysis of the absorbers under test.

EA—total deformation energy, absorbed during plastic deformation, expressed by the following formula:

(1) $$EA=\int_{0}^{x}F(x)dx,$$

where F(x) is the instantaneous crushing force and x is the specimen deformation.

SEA—the ratio of EA to the mass of the specimen under test, expressed by the following formula:

(2) SEA=EA/mE,

where m is the mass of the specimen under test.

MCF—mean crushing force for the known deformation length, expressed by the following formula:

(3) $$MCF=\frac{1}{x}\int_{0}^{x}F\left(x\right)dx,$$

PCF or highest crushing force is the maximum value of crushing force recorded during the test. By relating the PCF value to the MCF value, we can determine the CLE index, which refers to uniformity of load distribution over a structure [8,9].

(4) CLE=MCF/PCF

The results obtained were set against averaged coefficients for selected absorbers available on the market (UWWA) (Table 4).

At the highest impact velocity (24 km/h), all of the crash boxes under test produced CLE values higher than those generated by absorbers available on the market; still, it was WS-PPS that delivered the most advantageous results (a high MCF accompanied by a low PCF).

As far as peak crushing force (PCF index) is concerned, the highest value (considerably exceeding the results for the other specimens) was achieved by the WS-PEI absorber. This figure is ca. one-and-a-half times higher than the result obtained for the WS-PPS or UWWA absorbers.

As for the SEA index (defined as the ratio of EA to the mass of the specimen under test), the differences between the above absorbers are slight, but still, the WS-PPS specimen performed best. Compared with the crash boxes currently used in vehicles, the absorbers under test generated more advantageous SEA values, which is due to their relatively low mass. However, it needs to be borne in mind that in the case of absorbers used in vehicles, this index is of little importance (the mass of an absorber accounts for a small percentage of the mass of the entire vehicle).

**Figure 15:**
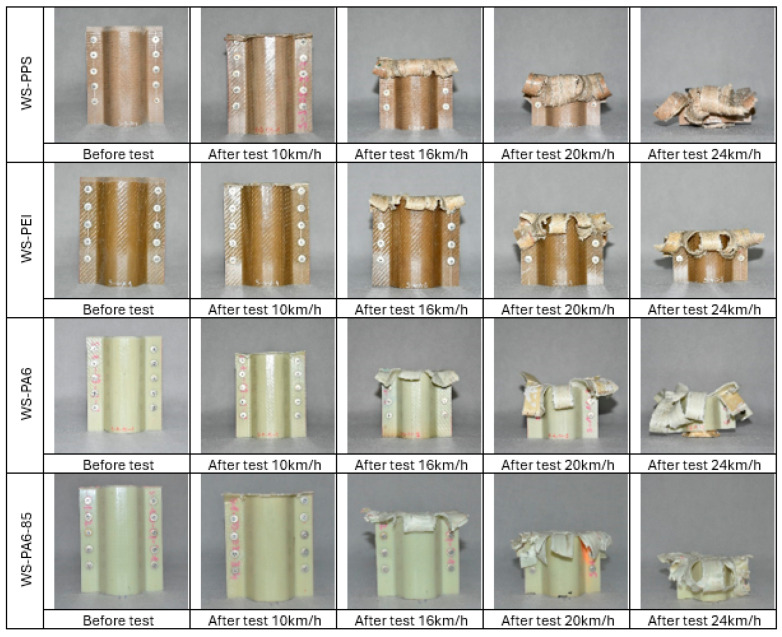
Crash boxes before and after tests.

A view of the structures before and after tests at each speed is shown in Figure 15. 

## 5. Conclusions

The tests conducted allowed the authors to determine the energy absorption capability of the crash boxes developed, particularly in terms of energy conversion irreversibility as well as their ability to keep force at a constant level during deformation. These properties are of key importance from the perspective of the use of such structures in the automotive industry.

An analysis of the restitution coefficients obtained yields a conclusion that all of the absorbers under test generated advantageous results, i.e., values close to zero, falling within the range of 0.05–0.18, which is evidence of the plastic nature of the deformations or the irreversibility of energy conversion. In respect of the speed of 10 km/h, the most advantageous result was generated by the WS-PA6 material. In the other cases, the best values were recorded for WS-PA6-85%.

The stability of the energy dissipation process is best illustrated by a waveform of deceleration as a function of deformation for speeds of 20 km/h and 24 km/h. The WS-PPS and WS-PA6 absorbers produced the most linear waveform for the deceleration of the test sled as a function of specimen deformation; i.e., after reaching the maximum deceleration value (or a value close to a maximum), this value did not change or changed slightly until the time the specimen was completely deformed, which demonstrates that the process of specimen deformation was stable. The WS-PEI absorber produced the least linear deceleration waveform.

Of all of the absorbers discussed, in the context of energy absorption, it was the WS-PPS absorber that produced the most advantageous results. The values of the coefficients for this absorber show that it is capable of absorbing the most energy per unit of mass (the highest SEA index—28.5 kJ/kg) and per unit of length (the highest MCF index—46.21 kJ/m). What distinguished this absorber was the lowest crushing force (PCF index—85 kN), which, if considered together with average crushing force, produced the most advantageous index of the uniformity of load over the structure (CLE index—0.54). It is advisable to use this material as a component of the energy-absorbing structure of a vehicle body, i.e., a crash box, whose function is to absorb impact energy at low speeds.

The authors are planning to continue their research relating to the development of an energy absorber for passenger cars, particularly in the context of the use of such a structure in vehicles.

## Figures and Tables

**Table 1:** Head-on collisions in Poland National Police Headquarters, Road Traffic Department, 2022.

Year	Accidents		Fatalities		Injured	
	Total	%	Total	%	Total	%
2022	2161	10.1	373	19.7	3132	12.7
2021	2489	10.9	433	19.3	3587	13.6
2020	2329	9.9	415	16.7	3277	12.4
2019	2938	9.7	585	20.1	4405	12.4
2018	3104	9.8	510	17.8	4676	12.5
2017	3172	9.7	507	17.9	4940	12.5
2016	3186	9.5	590	19.6	5002	12.3
2015	3049	9.2	511	17.4	4837	12.7

**Table 2:** Crash box specifications.

Description	Labelling	Dimensions			
		Width [mm]	Height [mm]	Length [mm]	Mass [g]
Glass-fibre-reinforced polyphenylene sulphide (PPS) absorber	WS-PPS	60	110	140	227
Glass-fibre-reinforced polyetherimide (PEI) absorber	WS-PEI	60	110	140	240
Glass-fibre-polyamide 6 (PA 6) absorber	WS-PA6	60	110	140	247
Glass-fibre-polyamide 6 (PA 6) absorber, with 85% of fibres running in one direction	WS-PA6-85%	60	110	140	239

**Table 3:** Restitution coefficients obtained.

Absorber Type	Parameter	10 km/h	16 km/h	20 km/h	24 km/h
WS-PPS	*k*	0.18	0.11	0.07	0.06
	% of energy	3.2%	1.2%	0.5%	0.3%
WS-PEI	*k*	0.29 *	0.12	0.11 *	0.08
	% of energy	8.1% *	1.5%	1.1% *	0.7%
WS-PA6	*k*	0.13	0.09	0.07	0.06
	% of energy	1.6%	0.9%	0.5%	0.3%
WS-PA6-85%	*k*	0.14	0.09	0.06	0.05
	% of energy	2.0%	0.8%	0.4%	0.3%

* During the test, in the restitution phase, the absorber slid out of the mounting on the sled. This is why the results of the restitution coefficient and energy calculations were affected by a gross error and should not be taken into consideration in further analysis.

**Table 4:** EA, PCF, SEA, MCF, and CLE indexes obtained for a speed of 24 km/h.

	Absorbed Energy [J]	PCF [kN]	SEA [kJ/kg]	MCF [kJ/m]	CLE
WS-PPS	6469	85	28.50	46.21	0.54
WS-PEI	6353	136	26.47	45.38	0.33
WS-PA6	6228	101	25.21	44.48	0.44
WS-PA6-85%	5902	108	24.69	42.16	0.39
UWWA	4099	94	4.01	27.29	0.31

## Data Availability

Data are contained within the article.

## References

[1] Polska ma Jedyną Szansę, aby Stać się Europejską Potęgą w Sektorze Nowej Mobilności. https://pspa.com.pl/2023/informacja/polska-ma-jedyna-szanse-aby-stac-sie-europejska-potega-w-sektorze-nowej-mobilnosci/.

[2] Jaździk-Osmólska A. (2022). Wycena Kosztów Wypadków i Kolizji Drogowych na Sieci Dróg w Polsce na Koniec 2021, z Wyodrębnieniem Średnich Kosztów Społeczno-Ekonomicznych Wypadków na Transeuropejskiej Sieci Transportowej.

[3] World Health Organization (2023). Global Status Report on Road Safety 2023.

[4] Komenda Główna Policji Biuro Ruchu Drogowego (2023). Wypadki Drogowe w Polsce w 2022 Roku.

[5] Wicher J. (2004). Bezpieczeństwo Samochodów i Ruchu Drogowego.

[6] Jackowski J., Łęgiewicz J., Wieczorek M. (2011). Samochody Osobowe i Pochodne.

[7] Khatri N.A., Shaikh H., Maher Z.A., Shah A., Ahmed S.F. (2018). A Review on Optimization of Vehicle Frontal Crashworthiness for Passenger Safety. Int. J. Eng. Technol..

[8] Wei W., Zhang F., Xing Y., Wang H., Liu R. (2023). Research on Mechanical Properties of Origami Aluminum Honeycomb for Automobile Energy Absorbing Box. Materials.

[9] Juntikka R., Hallström S. (2004). Selection of Energy Absorbing Materials for Automotive. Head Impact Countermeasures. Cell. Polym..

[10] Gidlewski M., Jackowski J., Posuniak P. (2022). Review and Analysis of Technical Designs of Rear Underrun Protective Devices (RUPDs) in Terms of Regulatory Compliance. Sensors.

[11] Mudassir M., Tarlochan F., Mansour M.A. (2020). Nature-Inspired Cellular Structure Design for Electric Vehicle Battery Compartment: Application to Crashworthiness. Appl. Sci..

[12] Tarlochan F. (2021). Sandwich Structures for Energy Absorption Applications: A Review. Materials.

[13] Ciampaglia A., Patruno L., Ciardiello R. (2024). Design of a Lightweight Origami Composite Crash Box: Experimental and Numerical Study on the Absorbed Energy in Frontal Impacts. J. Compos. Sci..

[14] Pirmohammad S., Marzdashti S.E. (2016). Crushing behavior of new designed multi-cell members subjected to axial and oblique quasi-static loads. Thin-Walled Struct..

[15] Santosa S., Wierzbicki T. (1998). Crash behavior of box columns filled with aluminum honeycomb or foam. Comput. Struct..

[16] Ahmad Z., Thambiratnam D. (2009). Dynamic computer simulation and energy absorption of foam-filled conical tubes under axial impact loading. Comput. Struct..

[17] Jackowski J., Posuniak P., Zielonka K., Jurecki R. (2023). Experimental Testing of Energy-Absorbing Structures Used to Enhance the Crashworthiness of the Vehicles. Energies.

[18] Jackowski J., Posuniak P., Rożnowicz Z. (2022). Experimental testing of selected energy absorbers in the context of their use in rear underrun protective devices. IOP Conference Series: Materials Science and Engineering.

[19] Lu G., Yu T. (2003). Energy Absorption of Structures and Materials.

[20] Abdulqadir S.F., Tarlochan F. (2022). Composite Hat Structure Design for Vehicle Safety: Potential Application to B-Pillar and Door Intrusion Beam. Materials.

[21] Seitzberger M., Rammerstorfer F.G., Degischer H.P., Gradinger R. (1997). Crushing of axially compressed steel tubes filled with aluminium foam. Acta Mech..

[22] Zhang Y., Ge P., Lu M., Lai X. (2018). Crashworthiness study for multi-cell composite filling structures. Int. J. Crashworthiness.

[23] Rogala M., Gajewski J., Ferdynus M. (2020). The Effect of Geometrical Non-Linearity on the Crashworthiness of Thin-Walled Conical Energy-Absorbers. Materials.

